# Cathelicidin antimicrobial protein, vitamin D, and risk of death in critically ill patients

**DOI:** 10.1186/s13054-015-0812-1

**Published:** 2015-03-10

**Authors:** David E Leaf, Heather E Croy, Sara J Abrahams, Anas Raed, Sushrut S Waikar

**Affiliations:** Division of Renal Medicine, Brigham and Women’s Hospital, 75 Francis Street, Boston, MA 02115 USA

## Abstract

**Introduction:**

Decreased production of cathelicidin antimicrobial protein-18 (hCAP18) has been proposed to be a key mechanism linking decreased 25-hydroxyvitamin D (25D) levels with adverse outcomes among critically ill patients. However, few studies in humans have directly assessed plasma hCAP18 levels, and no study has evaluated the association between hCAP18 levels and adverse outcomes among critically ill patients.

**Methods:**

We performed a single-center, prospective cohort study among 121 critically ill patients admitted to intensive care units (ICUs) between 2008 and 2012. We measured plasma hCAP18, 25D, D-binding protein, and parathyroid hormone levels on ICU day 1. The primary endpoint was 90-day mortality. Secondary endpoints included hospital mortality, sepsis, acute kidney injury, duration of mechanical ventilation, and hospital length of stay.

**Results:**

ICU day 1 hCAP18 levels were directly correlated with 25D levels (Spearman’s rho (r_s_) = 0.30, *P* = 0.001). In multivariate analyses adjusted for age and Acute Physiology and Chronic Health Evaluation II (APACHE II) score, patients with hCAP18 levels in the lowest compared to highest tertile on ICU day 1 had a 4.49 (1.08 to 18.67) greater odds of 90-day mortality, and also had greater odds of sepsis. ICU day 1 levels of other analytes were not associated with 90-day mortality.

**Conclusions:**

Lower 25D levels on ICU day 1 are associated with lower hCAP18 levels, which are in turn associated with a greater risk of 90-day mortality. These findings provide a potential mechanistic basis for the frequently observed association between low 25D levels and poor outcomes in critically ill patients.

**Electronic supplementary material:**

The online version of this article (doi:10.1186/s13054-015-0812-1) contains supplementary material, which is available to authorized users.

## Introduction

Vitamin D deficiency/insufficiency is very common in critically ill patients, with reported rates ranging from 81.5% to 99% [1-3]. Further, multiple studies have documented associations between decreased 25-hydroxyvitamin D (25D) levels and increased risk of adverse outcomes including prolonged length of stay [3], infection [4], acute kidney injury (AKI) [5], and mortality [3-7]. These associations are often attributed to the effects of vitamin D metabolites on host defense, which have been well characterized *in vitro* [8-10]. However, few studies in humans have simultaneously evaluated 25D levels together with markers of host defense in critically ill patients.

The strongest known link between vitamin D metabolites and the immune system relates to the effects of vitamin D metabolites on the production of cathelicidins [10], a critical family of antimicrobial proteins. Cathelicidins act by disrupting foreign-cell membranes, binding lipopolysaccharide residues, and recruiting leukocytes [11,12]. In animal models, deficiency of cathelicidin is associated with increased susceptibility to bacterial infection [13-15], whereas overexpression confers protection [16]. In humans, cathelicidin antimicrobial protein-18 (hCAP18) is the only known cathelicidin. hCAP18 is found primarily in the granules of neutrophils, and is also produced and secreted by monocytes, macrophages, epithelial cells, and other cell types [17]. Low 25D levels are associated with reduced production of hCAP18 by macrophages infected with *Mycobacterium tuberculosis*, whereas treatment with the activated vitamin D metabolite 1,25-dihydroxyvitamin D (1,25D) *in vitro* results in enhanced production of hCAP18 and improved killing of the microorganisms [10]. In addition to macrophages, the *in vitro* inducibility of hCAP18 by 25D and 1,25D has also been demonstrated in multiple other human cell lines [10,18-22].

Despite the strong link between vitamin D metabolites and hCAP18 demonstrated in preclinical models, no study to our knowledge has measured plasma hCAP18 levels and evaluated their association with adverse outcomes in critically ill patients. Additionally, conflicting results have been reported on the association between plasma hCAP18 and 25D levels in humans [23-25]. We hypothesized that 25D and hCAP18 levels are directly correlated in critically ill patients, and that lower hCAP18 levels are associated with increased risk of 90-day mortality.

## Materials and methods

### Study design

We conducted a prospective cohort study among patients admitted to intensive care units (ICUs) at Brigham and Women’s Hospital between 2008 and 2012. Patients or their surrogates provided written informed consent and all protocols were approved by the Partners Human Research Committee (protocol #2007P000894), which is the Institutional Review Board for Brigham and Women’s Hospital.

Inclusion criteria were age ≥18 years and admission to a medical or surgical ICU. Exclusion criteria were: (1) anticipated ICU stay <24 hours; (2) admitted to the ICU for a low-risk condition such as airway monitoring; (3) serum creatinine >4.5 mg/dl or receiving dialysis; (4) pregnancy; and (5) institutionalized individuals.

We collected venous blood samples daily on ICU days 1 through 5. Blood samples were collected into EDTA-containing vacutainers, centrifuged at 3,200 RPM for 15 minutes, and the plasma was aliquoted and stored at −80°C within 2 hours of collection. We measured analytes in plasma samples at two time points: within 24 hours of ICU arrival and 48 hours later (hereafter referred to as ICU day 1 and 3, respectively).

### Clinical outcomes

Investigator DEL adjudicated all outcomes by reviewing discharge summaries and progress notes, and was blinded to all study measurements at the time of adjudication. The primary endpoint was 90-day mortality. Secondary endpoints were hospital mortality, sepsis, incident AKI, duration of mechanical ventilation, and hospital length of stay. The association between hCAP18 levels and sepsis was assessed cross-sectionally since many of the patients already met sepsis criteria upon arrival to the ICU. Other outcomes were assessed prospectively.

Sepsis was defined according to consensus definition [26]. Incident AKI was defined according to serum creatinine-based criteria established by the Kidney Disease Improving Global Outcomes Work Group [27]. Patients who already had AKI (N = 4) on arrival to the ICU were excluded from analyses of incident AKI. Duration of mechanical ventilation and hospital length of stay were assessed using ventilator-free days and hospital-free days to avoid the confounding effect of mortality. Ventilator-free days and hospital-free days were defined as 28 minus the number of ventilator-dependent days or hospitalization days, respectively, assuming survival to 28 days or discharge from the hospital. Patients who died before 28 days were assigned a score of zero [28,29]. In exploratory analyses, we also assessed whether hCAP18 levels on ICU day 1 differed by sepsis severity, primary type of infection, or primary organism.

### Laboratory measurements

All biomarkers were measured on ICU days 1 and 3 except parathyroid hormone (PTH), which was measured on ICU day 1 only. Assays were performed at the Harvard Medical School Clinical and Translational Science Award core laboratory. Plasma hCAP18 levels were measured by enzyme-linked immunosorbent assay (ELISA) using a commercially available kit (Hycult Biotech, Uden, Netherlands) which recognizes the 37 amino acid biologically active C-terminal fragment (LL-37) cleaved from hCAP18 [30]. Plasma 25D (combined D_2_ and D_3_, hereafter referred to as ‘total 25D’) and D-binding protein (DBP) levels were measured by ELISA using commercially available kits (Abbott Laboratories, Abbott Park, IL, USA and R&D Systems, Inc., Minneapolis, MN, USA, respectively). Plasma intact PTH was measured using a chemiluminescent immunoassay (Beckman Coulter, Fullerton, CA, USA). Interassay coefficients of variation, estimated using blinded replicate samples from ICU patients, were 5.5% for hCAP18, 3.5% for total 25D, 11% for DBP, and 3.1% for PTH.

### Bioavailable and free 25D

Because total 25D circulates bound to DBP (85 to 90%) and albumin (10 to 15%), less than 1% of circulating hormone exists in its free form [31]. The sum of free- and albumin-bound hormone is often referred to as ‘bioavailable’, because 25D bound to DBP is thought to have limited biological activity. In the absence of commercially available assays to directly measure bioavailable and free 25D levels, most studies have relied on formulas that incorporate binding coefficients for DBP and albumin, coupled to measurement of total 25D, DBP, and albumin levels [31-33]. Using these formulas, bioavailable compared to total 25D levels are more strongly associated with bone mineral density among young healthy adults [32]; serum calcium and PTH levels among incident hemodialysis patients [34]; and mortality among hospitalized patients [35]. We therefore used these equations to estimate bioavailable and free 25D levels. The equations used to calculate these levels are provided in Additional file 1.

### Statistical analyses

Statistical analysis was performed with SAS Version 9.3 (SAS Institute Inc., Cary, NC, USA). Correlations between hCAP18 levels with total 25D, bioavailable 25D, free 25D, and PTH levels on ICU day 1 were assessed using Spearman’s rank correlation coefficient. Comparison of biomarker levels between patients who were alive vs. dead at 90 days was assessed using the Wilcoxon rank sum test. Comparison of biomarker levels over time (ICU day 1 vs. day 3) was assessed using the Wilcoxon signed-rank test. Differences in hCAP18 levels on ICU day 1 on the basis of sepsis severity, primary type of infection, and primary organism were assessed using the Kruskal-Wallis test.

Logistic regression was used to assess the association between tertiles of biomarker levels on ICU day 1 and 90-day mortality (the primary outcome) as well as hospital mortality, sepsis, and AKI (secondary outcomes). The highest tertile served as the reference group. Multivariable models included age and acute physiology and chronic health evaluation (APACHE II) score, calculated on ICU day 1. In exploratory analyses we also adjusted for ICU day 1 estimated glomerular filtration rate (eGFR), calculated using the chronic kidney disease epidemiology equation [36], since reduced kidney function could affect hCAP18 levels. Tertiles of biomarker levels on ICU day 1 and 90-day mortality were also assessed in aggregate using the Cochran-Armitage test for trend. The association between hCAP18 levels on ICU day 1 and ventilator-free days and hospital-free days was assessed using Spearman’s rank correlation coefficient. All comparisons are two-tailed, with *P* <0.05 considered significant.

## Results

We enrolled and collected samples from 121 patients. Median (interquartile range (IQR)) age was 62 (56 to 73) years. The most common comorbidities were hypertension (53%), chronic lung disease (31%), and active malignancy (28%). The majority of patients (84%) were admitted to a surgical ICU. Additional baseline characteristics are shown in Table 1.

**Table 1:** **Baseline characteristics**

	**N = 121**
Demographics	
Age (yr) - median (IQR)	62 (56-73)
Female sex (%)	46 (38)
White race (%)	112 (93)
Comorbidities (%)	
Hypertension	64 (53)
Chronic lung disease	37 (31)
Active malignancy	34 (28)
Diabetes mellitus	29 (24)
Chronic kidney disease	7 (6)
Congestive heart failure	4 (3)
Chronic liver disease	2 (2)
Severity of illness	
APACHE II score - median (IQR)*	14 (10-16)
Charlson Comorbidity Index - median (IQR)^†^	2 (1-4)
ICU type	
Surgical	102 (84)
SICU	66 (54)
TICU	36 (30)
Nonsurgical	19 (16)
CCU	11 (9)
MICU	8 (7)
Status of procedure (for surgical patients) (%)	
Elective	41 (34)
Urgent	57 (47)
Days in hospital prior to enrollment – median (IQR)	1 (1-3)

*APACHE II, Acute Physiology and Chronic Health Evaluation II, is an ICU severity of illness scoring system ranging from 0 to 71, with higher scores corresponding to more severe disease; ^†^Charlson Comorbidity Index is a prognostic tool that has been validated in critically ill patients, with higher scores indicating worse prognosis [37]. IQR, interquartile range; ICU, intensive care unit; SICU, surgical/trauma ICU; TICU, thoracic ICU; CCU, cardiac care unit; MICU, medical ICU.

### hCAP18 and vitamin D markers

Table 2 shows plasma levels of hCAP18, total 25D, bioavailable 25D, and free 25D, DBP, albumin, and PTH from ICU days 1 and 3. Levels of total 25D on ICU day 1 were <20 ng/ml in 68% of patients and <30 ng/ml in 95% of patients. Other than albumin, which declined over time, plasma levels of other biomarkers were unchanged between ICU days 1 and 3.

**Table 2:** **hCAP18 and mineral metabolite/hormone levels on ICU days 1 and 3**

**Biomarker**	**Reference range**	**ICU day 1 (N = 121)**	**ICU day 3 ** **(N = 111)** ^**†**^	***P*** **value**
**hCAP18 (ng/ml)**	Undefined	160 (106-286)	166 (116-291)	0.92
**Total 25D (ng/ml)**	30-80	17 (13-22)	17 (14-21)	0.42
**Bioavailable 25D (ng/ml)**	Undefined	2.5 (1.8-3.5)	2.4 (1.6-3.3)	0.24
**Free 25D (pg/ml)**	Undefined	9.6 (6.5-12.9)	9.5 (6.5-12.9)	0.98
**DBP (mg/dl)**	20-55	12.7 (9.4-17.1)	13.7 (10.1-17.1)	0.27
**Albumin (g/dl)**	4.1-5.3	3.1 (2.4-3.5)	2.9 (2.3-3.3)	0.002
**PTH (pg/ml)**	15-65	40 (28-56)	---	N/A

Values represent median (25^th^ to 75^th^ interquartile range). ^†^Fewer samples were available on ICU day 3 due to interim death or discharge. ICU, intensive care unit; hCAP18, human cathelicidin antimicrobial protein-18; 25D, 25-hydroxyvitamin D; DBP, D-binding protein; PTH, parathyroid hormone.

### Factors associated with hCAP18 levels

**Figure 1:**
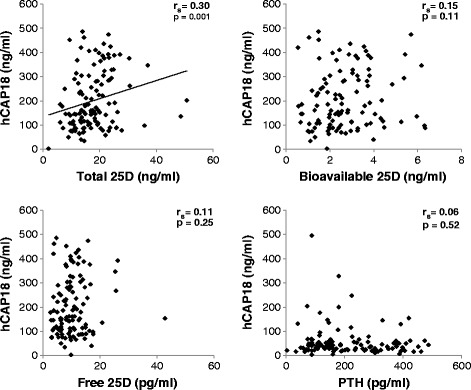
**Correlations between plasma hCAP18**, **25D metabolites, and PTH.** Total 25D represents the sum of all 25-hydroxyvitamin D (both D_2_ and D_3_), including 25D bound to DBP, albumin, and circulating freely; bioavailable 25D is the sum of albumin-bound and free 25D; free 25D is the circulating fraction of 25D that is neither bound to DBP nor albumin. DBP, D-binding protein; hCAP18, human cathelicidin antimicrobial protein-18; PTH, parathyroid hormone.

Plasma hCAP18 levels on ICU day 1 correlated positively with total 25D (Spearman’s rho (r_s_) = 0.30, *P* = 0.001) but not with bioavailable 25D, free 25D, or PTH levels (Figure 1). Plasma hCAP18 levels on ICU day 1 did not differ by age, gender, type of ICU (surgical vs. nonsurgical), or status of procedure (urgent vs. elective). Similarly, plasma hCAP18 levels on ICU day 1 did not differ by comorbidities except for active malignancy (median (IQR) 120 (78 to 174) and 186 (116 to 304) ng/ml for patients with vs. without active malignancy, *P* = 0.002). Additionally, we found no correlation between hCAP18 levels and eGFR on ICU day 1 (Additional file 2).

### hCAP18 and adverse outcomes

Among the 121 participants, 71 (59%) had sepsis, 9 (7%) died in the hospital, and 21 (17%) died within 90 days. Data on sepsis severity, primary site of infection, and primary organism for the 71 patients who developed sepsis are shown in Table 3. Plasma hCAP18 levels on ICU day 1 did not differ by sepsis severity (*P* = 0.14), primary site of infection (*P* = 0.23), or primary organism (*P* = 0.07) (Additional file 3).

**Table 3:** **Sepsis characteristics**

	**N = 71**
**Sepsis severity**	
Sepsis	24 (34)
Severe sepsis	30 (42)
Septic shock	17 (24)
**Primary site of infection**	
Respiratory	35 (49)
Abscess or necrotizing fasciitis	13 (18)
Gastrointestinal/peritonitis	12 (17)
Unknown or other^†^	11 (16)
**Primary organism**	
*Staphylococcus aureus*	14 (20)
Other Gram(+)^‡^	13 (18)
*Pseudomonas*, *Klebsiella*, *Escherichia coli*, and *Enterobacter*	15 (21)
Other Gram(−)^¶^	8 (11)
Unknown or other^§^	21 (30)

Values represent *n* (%). ^†^Includes bacteremia (N = 5), urosepsis (N = 2), pericarditis (N = 1), and septic arthritis (N = 1); ^‡^includes *Enterococus* (N = 7), *Lactobacillus* (N = 2), coagulase-negative *Staphylococcus* (N = 2), *Streptococcus pneumoniae* (N = 1), and *Streptococcus mitis* (N = 1); ^¶^includes *Proteus mirabilis* (N = 2), *Serratia* (N = 2), *Citrobacter* (N = 1), *Moraxella catarrhalis* (N = 1), *Fusobacterium necrophorum* (N = 1), and *Hemophilus influenza* (N = 1); ^§^includes *Clostridium difficile* (N = 3) and *Aspergillus* (N = 1).

**Figure 2:**
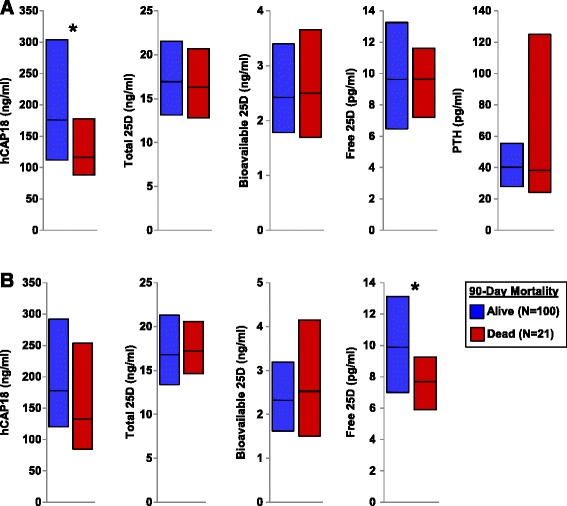
**Plasma hCAP18, 25D metabolites, and PTH levels among survivors vs. nonsurvivors at 90 days. (A)** Biomarker levels on ICU day 1. **(B)** Biomarker levels on ICU day 3. ^*^ *P* <0.05, comparison of biomarker levels among survivors vs. nonsurvivors. Bars represent median (25^th^ to 75^th^ interquartile range). 25D, 25-hydroxyvitamin D; hCAP18, human cathelicidin antimicrobial protein-18; ICU, intensive care unit; PTH, parathyroid hormone.

Biomarker levels on ICU days 1 and 3 in patients who were alive vs. dead at 90 days are shown in Figure 2. Plasma hCAP18 levels on ICU day 1 were lower in patients who died compared to patients who survived (median (IQR) 116 (88 to 178) and 176 (112 to 304) ng/ml, *P* = 0.04). Additionally, free 25D levels on ICU day 3 were lower in patients who died compared to patients who survived (median (IQR) 7.7 (5.9 to 9.3) and 9.9 (7.0 to 13.1), *P* = 0.03). Other biomarker levels were similar between survivors vs. nonsurvivors.

**Figure 3:**
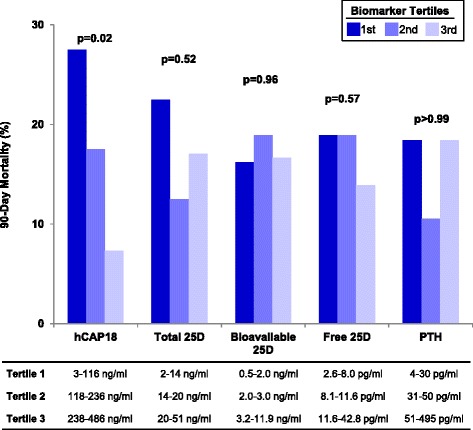
**Tertiles of plasma hCAP18, 25D metabolites, and PTH on ICU day 1 and risk of 90-day mortality.** 25D, 25-hydroxyvitamin D; hCAP18, human cathelicidin antimicrobial protein-18; ICU, intensive care unit; PTH, parathyroid hormone.

In univariate analyses, patients with ICU day 1 hCAP18 levels in the lowest vs. highest tertile had a significantly greater risk of 90-day mortality (Table 4). After adjusting for age and APACHE II score, the association was slightly attenuated but remained significant (adjusted odds ratio = 4.49, 95% confidence interval = 1.08 to 18.67, *P* = 0.04). In exploratory analyses, further adjustment for ICU day 1 eGFR did not affect the association (adjusted odds ratio = 4.44, 95% confidence interval = 1.07 to 18.45, *P* = 0.04). Tertiles of other biomarker levels on ICU day 1 were not associated with 90-day mortality (Table 4). We found similar findings when we analyzed the data using tests of trend across all three tertiles (Figure 3).

**Table 4:** **Unadjusted and multivariable-adjusted odds ratios comparing ICU day 1 levels of hCAP18, 25D metabolites, and PTH and risk of 90-day mortality**

**Outcome**	**Unadjusted**		**Adjusted**	
	**Odds ratio (95% CI)**	***P*** **value**	**Odds ratio (95% CI)**	***P*** **value**
**hCAP18**				
Tertile 1	4.80 (1.23-18.80)	0.02	4.49 (1.08-18.67)	0.04
Tertile 2	2.69 (0.64-11.23)	0.18	3.00 (0.67-13.25)	0.15
Tertile 3 (REF)	1.00	N/A	1.00	N/A
**Total 25D**				
Tertile 1	1.41 (0.47-4.24)	0.54	1.39 (0.41-4.73)	0.60
Tertile 2	0.69 (0.21-2.40)	0.56	0.69 (0.19-2.56)	0.58
Tertile 3 (REF)	1.00	N/A	1.00	N/A
**Bioavailable 25D**				
Tertile 1	0.97 (0.28-3.34)	0.96	1.11 (0.28-4.36)	0.88
Tertile 2	1.17 (0.35-3.88)	0.80	1.39 (0.36-5.34)	0.63
Tertile 3 (REF)	1.00	N/A	1.00	N/A
**Free 25D**				
Tertile 1	1.45 (0.41-5.06)	0.56	1.47 (0.37-5.82)	0.59
Tertile 2	1.45 (0.41-5.06)	0.56	1.88 (0.44-8.05)	0.40
Tertile 3 (REF)	1.00	N/A	1.00	N/A
**PTH**				
Tertile 1	1.00 (0.31-3.19)	>0.99	1.22 (0.36-4.13)	0.75
Tertile 2	0.52 (0.14-1.95)	0.33	0.57 (0.15-2.25)	0.43
Tertile 3 (REF)	1.00	N/A	1.00	N/A

Twenty-one events. Adjusted models include age and APACHE II score. ICU, intensive care unit; hCAP18, human cathelicidin antimicrobial protein-18; 25D, 25-hydroxyvitamin D; PTH, parathyroid hormone; CI, confidence interval; APACHE II, acute physiology and chronic health evaluation II.

Table 5 shows the unadjusted and multivariable-adjusted odds ratios evaluating the associations between ICU day 1 hCAP18 levels and hospital mortality, sepsis, and incident AKI. We found significant associations between hCAP18 levels in the lowest and middle tertiles and sepsis. We found no association between hCAP18 tertiles and hospital mortality or incident AKI.

**Table 5:** **Unadjusted and multivariable-adjusted odds ratios comparing ICU day 1 hCAP18 levels and secondary outcomes**

**Outcome**	**Unadjusted**		**Adjusted**	
	**Odds ratio (95% CI)**	***P*** **value**	**Odds ratio (95% CI)**	***P*** **value**
**Hospital mortality (9 events)**				
Tertile 1	2.17 (0.37-12.55)	0.39	1.97 (0.33-11.75)	0.46
Tertile 2	1.58 (0.25-10.00)	0.63	1.66 (0.26-10.78)	0.60
Tertile 3 (REF)	1.00	N/A	1.00	N/A
**Sepsis (71 events)**				
Tertile 1	2.35 (0.96-5.74)	0.06	2.54 (1.01-6.4)	0.047
Tertile 2	3.72 (1.47-9.45)	0.006	3.64 (1.40-9.44)	0.008
Tertile 3 (REF)	1.00	N/A	1.00	N/A
**AKI (33 events)**				
Tertile 1	0.65 (0.22-1.96)	0.45	0.61 (0.20-1.88)	0.39
Tertile 2	2.06 (0.78-5.45)	0.15	2.01 (0.74-5.46)	0.17
Tertile 3 (REF)	1.00	N/A	1.00	N/A

Adjusted models include age and APACHE II score. ICU, intensive care unit; hCAP18, human cathelicidin antimicrobial protein-18; CI, confidence interval; AKI, acute kidney injury; APACHE II, Acute Physiology and Chronic Health Evaluation II.

Finally, we found no correlation between ICU day 1 hCAP18 levels and ventilator-free days (r_s_ = −0.01, *P* = 0.96). We found a nonsignificant trend between hCAP18 levels and hospital-free days (r_s_ = 0.16, *P* = 0.06), implying a trend toward decreased hospital length of stay in patients with higher hCAP18 levels.

## Discussion

In this prospective cohort study among critically ill patients, we found that lower total 25D levels were associated with lower hCAP18 levels, which were in turn associated with a greater risk of 90-day mortality. These findings provide a potential mechanistic basis for the commonly observed association between low 25D levels and poor outcomes in critically ill patients.

The current findings expand on prior studies of vitamin D metabolites and hCAP18. Preclinical studies have demonstrated potent antimicrobial effects of hCAP18 against a broad range of pathogens [13-16,38-43]. Further, the *in vitro* inducibility of hCAP18 by 25D and 1,25D has been demonstrated in multiple human cell lines [10,18-22]. These preclinical studies are complemented by numerous observational studies in humans, which have consistently shown associations between decreased 25D levels and increased mortality in patients with critical illness [3-7] and in other settings [44-46]. The association between decreased 25D levels and adverse outcomes in critical illness has been postulated to be mediated, at least in part, by decreased production of hCAP18 [6,47,48], yet few studies have simultaneously measured 25D and hCAP18 and evaluated their association with adverse outcomes. None, to our knowledge, have done so in critically ill patients.

hCAP18 levels have been reported in critically ill patients in only two other studies, neither of which reported clinical outcomes. Jeng *et al*. found lower hCAP18 levels in critically ill patients (N = 49) compared to healthy controls (N = 21) [25]. In a larger study (N = 130), no difference in hCAP18 levels was detected among ICU patients with vs. without sepsis [49]. A third study, which was conducted among hemodialysis outpatients, reported an inverse association between hCAP18 levels and 1-year infection-related mortality [24]. Consistent with these findings, we found associations between lower hCAP18 levels with both sepsis and 90-day mortality. Our findings raise the possibility that inadequate circulating levels of hCAP18, possibly due to decreased 25D levels, may predispose patients to infection and death. Consequently, randomized controlled trials (RCTs) are warranted to test whether administration of vitamin D metabolites may increase hCAP18 levels and thereby improve outcomes in critical illness.

A recent RCT tested the effect of 540,000 IU of vitamin D_3_ in 492 critically ill patients [50]. Although the primary outcome of hospital length of stay was negative, a prespecified analysis of patients with severe vitamin D deficiency, defined as 25D levels ≤12 ng/ml, found an almost 50% reduction in hospital mortality in the active treatment group compared to placebo. hCAP18 levels were not reported. In a pilot RCT of 67 critically ill patients with severe sepsis, we found that a single dose of calcitriol (1,25D) increased hCAP18 leukocyte mRNA expression at 24 hours but did not increase plasma levels [51]. Thus, additional RCTs are needed to evaluate whether hCAP18 is inducible in humans in response to vitamin D metabolites, as it is *in vitro*, and whether upregulating hCAP18 improves outcomes.

We acknowledge the limitations of this study including single-center, a maximum of two data points per patient, and observational design. Although we measured DBP and PTH, we did not measure all potentially relevant markers of mineral metabolism such as 1,25D and fibroblast growth factor-23, nor did we measure all potentially relevant markers of innate immunity, such as β defensin 2, which may also be upregulated by vitamin D metabolites [17]. Levels of bioavailable and free 25D were estimated using equations that were not developed and validated in critical illness. Therefore, estimated values for these vitamin D metabolites should be viewed as preliminary. Additionally, the association between hCAP18 levels on ICU day 1 and 90-day mortality could be confounded by a number of factors, such as severity of illness or comorbidities. Importantly, this association remained significant after adjusting for age and APACHE II score. Nonetheless, residual confounding from other variables cannot be excluded.

The association between hCAP18 levels and sepsis should be viewed cautiously for a variety of reasons. First, we did not have hCAP18 levels prior to the onset of acute illness and therefore cannot exclude the possibility of reverse causality, since sepsis was assessed cross-sectionally. Second, we cannot exclude the possibility of a type 1 error due to multiple comparisons. Finally, although approximately half of all sepsis cases were due to a respiratory infection, we did not find an association between hCAP18 levels and ventilator-free days, further suggesting that our finding of an association between hCAP18 levels and sepsis should be viewed as preliminary and in need of confirmation.

## Conclusions

In conclusion, our central findings are that lower total 25D levels on ICU day 1 are associated with lower hCAP18 levels, which are in turn associated with a greater risk of 90-day mortality. These data support the notion that vitamin D metabolites may have important effects on innate immunity via induction of hCAP18. RCTs are needed to determine whether vitamin D metabolites upregulate hCAP18 in humans, as they do *in vitro*, and whether such a strategy will improve outcomes among critically ill patients.

## Key messages

- hCAP18 levels on ICU day 1 correlate positively with total, but not bioavailable or free 25-hydroxyvitamin D levels.
- Lower hCAP18 levels on ICU day 1 are associated with increased risk of 90-day mortality.
- Vitamin D metabolites may have important effects on innate immunity via induction of hCAP18.

## Additional files

Additional file 1:
**Supplemental methods.** Calculation of bioavailable and free 25-hydroxyvitamin D levels. Additional file 2:
**Plasma hCAP18 levels on ICU day 1 and eGFR.** Plasma hCAP18 levels on ICU day 1 showed no correlation with eGFR. Additional file 3:
**Plasma hCAP18 levels on ICU day 1 by (A) sepsis severity, (B) primary site of infection, and (C) primary type of organism.**

## Abbreviations

1,25D: 1,25-dihydroxyvitamin D
25D: 25-hydroxyvitamin D
AKI: acute kidney injury
APACHE II: acute physiology and chronic health evaluation II
DBP: D-binding protein
eGRF: estimated glomerular filtration rate
ELISA: enzyme-linked immunosorbent assay
hCAP18: human cathelicidin antimicrobial protein-18
ICU: intensive care unit
IQR: interquartile range
PTH: parathyroid hormone
RCT: randomized controlled trial
R_s_: Spearman’s rho
