# Supplementary material for: Cathelicidin antimicrobial protein, vitamin D, and risk of death in critically ill patients
Source: Crit Care. 2015 Mar 10;19(1):80. doi: 10.1186/s13054-015-0812-1 (PMC4357206; doi:10.1186/s13054-015-0812-1)
Supplement: Additional file 1: — Supplemental methods. Calculation of bioavailable and free 25-hydroxyvitamin D levels. [file 13054_2015_812_MOESM1_ESM.pdf]

Supplemental Methods: Calculation of bioavailable- and free 25-hydroxyvitamin D levels

$[D_{total}]$ = total measured vitamin D concentration (either 25(OH)D or 1,25$(OH)_2$D)
[Alb] = measured albumin concentration
[DBP] = measured vitamin D binding protein concentration
$[D_{Alb}]$ = concentration of albumin-bound vitamin D
$[D_{DBP}]$ = concentration of DBP-bound vitamin D
$[D_{free}]$ = concentration of free (unbound) D
$[D_{bioavailable}]$ = concentration of Bioavailable D = $[D_{free}] + [D_{Alb}]$

K_{alb} = affinity constant between vitamin D and albumin = $6 \times 10^5\ M^{-1}$ (for 25-OH D) or $5.4 \times 10^4\ M^{-1}$ (for 1,25-OH2 D)

K_{DBP} = affinity constant between vitamin D and DBP = $7 \times 10^8\ M^{-1}$ (for 25-OH D) or $3.7 \times 10^7\ M^{-1}$ (for 1,25-OH2 D)

a = $K_{DBP} \cdot K_{alb} \cdot [Alb] + K_{DBP}$
b = $K_{DBP} \cdot [DBP] - K_{DBP} \cdot [D_{total}] + K_{alb} \cdot [Alb] + 1$
c = $-[D_{total}]$

$[D_{free}] = [-b + \sqrt{b^2 - 4ac}] \div 2a$

$[D_{bioavailable}] = [D_{free}] + [D_{Alb}] = (K_{alb} \cdot [Alb] + 1) \cdot [D_{free}]$
