# Supplementary material for: Cathelicidin antimicrobial protein, vitamin D, and risk of death in critically ill patients
Source: Crit Care. 2015 Mar 10;19(1):80. doi: 10.1186/s13054-015-0812-1 (PMC4357206; doi:10.1186/s13054-015-0812-1)
Supplement: Additional file 2: — Plasma hCAP18 levels on ICU day 1 and eGFR. Plasma hCAP18 levels on ICU day 1 showed no correlation with eGFR. [file 13054_2015_812_MOESM2_ESM.pdf]

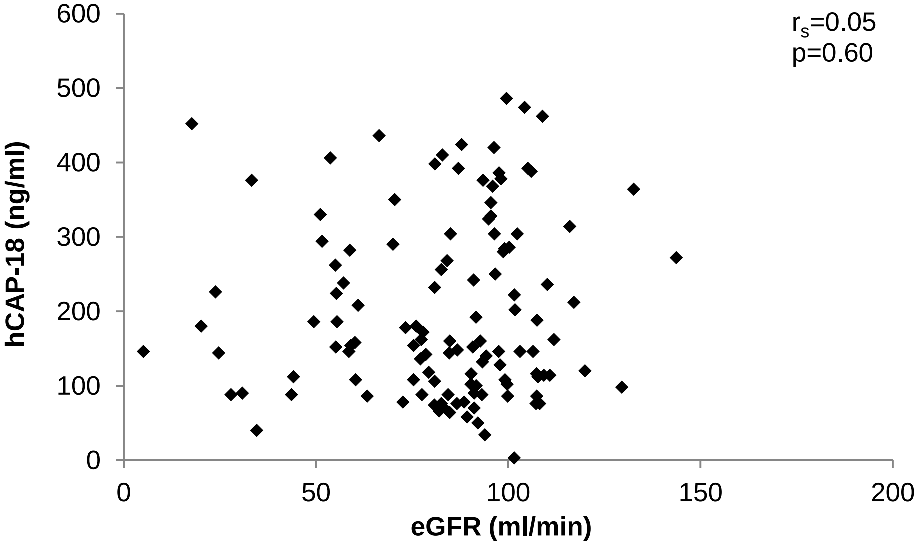

Additional File 2. Plasma hCAP18 levels on ICU Day 1 and eGFR. Plasma hCAP18 levels on ICU Day 1 showed no correlation with eGFR. eGFR=estimated Glomerular Filtration Rate (calculated using the Chronic Kidney Disease Epidemiology Collaboration (CKD-EPI) equation.
