# Supplementary material for: Cathelicidin antimicrobial protein, vitamin D, and risk of death in critically ill patients
Source: Crit Care. 2015 Mar 10;19(1):80. doi: 10.1186/s13054-015-0812-1 (PMC4357206; doi:10.1186/s13054-015-0812-1)
Supplement: Additional file 3: — Plasma hCAP18 levels on ICU day 1 by (A) sepsis severity, (B) primary site of infection, and (C) primary type of organism. [file 13054_2015_812_MOESM3_ESM.pdf]

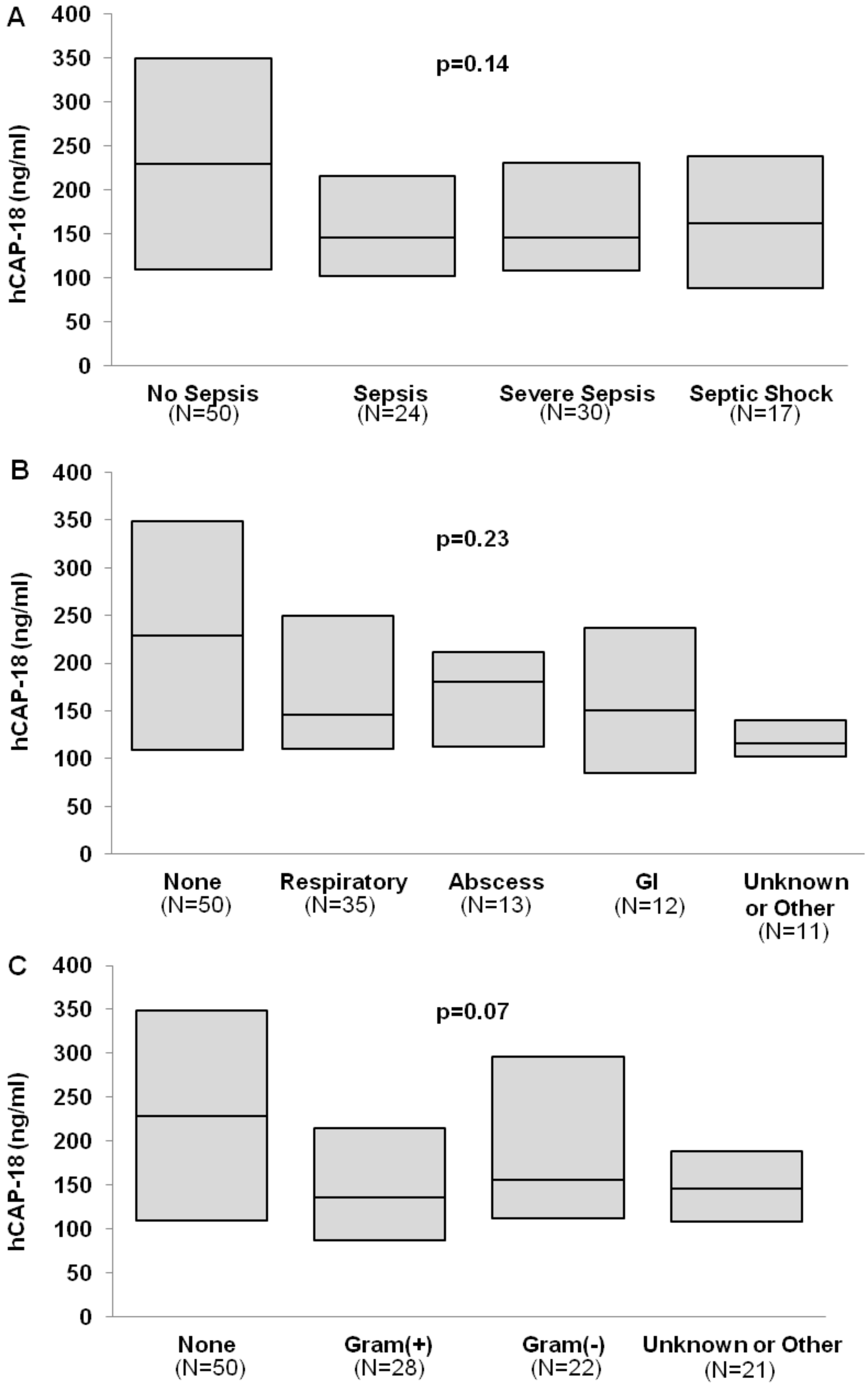

Additional File 3. Plasma hCAP18 levels on ICU Day 1 by (A) sepsis severity, (B) primary site of infection, and (C) primary type of organism.
